# Time-Dependent Adhesion and Fluoride Release of Resin-Modified Glass Ionomer Cements on Demineralized Enamel, Sound Enamel and Dentine

**DOI:** 10.3390/jcm14207166

**Published:** 2025-10-11

**Authors:** Pilar Cereceda-Villaescusa, Pilar Valverde-Rubio, Inmaculada Cabello, Amparo Pérez-Silva, Yolanda Martínez-Beneyto, Inmaculada Gómez Ríos, Antonio José Ortiz-Ruiz

**Affiliations:** 1 Department of Integrated Pediatric Dentistry, Faculty of Medicine, University of Murcia, 30100 Murcia, Spain; pilar.cerecedav@um.es (P.C.-V.); perez_amparo@hotmail.com (A.P.-S.); yolandam@um.es (Y.M.-B.); ajortiz@um.es (A.J.O.-R.); 2 Department of Integrated Pediatric Dentistry, Faculty of Medicine, Universidad Católica San Antonio de Murcia, Guadalupe, 30107 Murcia, Spain; mpvalverde@ucam.edu; 3 Department of Integral Paediatric Dentistry, School of Dentistry, University of Granada, 18071 Granada, Spain; icabello@ugr.es; 4 Biomedical Research Institute of Murcia Pascual Parilla-IMIB, 30120 Murcia, Spain

**Keywords:** shear strength, resin-modified glass ionomer cement, field emission scanning electron microscopy, FESEM, fluoride ion

## Abstract

**Background:** The treatment of cavitated lesions has evolved with minimally invasive dentistry (MID), whereby we can leave demineralized enamel that could potentially be remineralizable with the use of materials such as resin-modified glass ionomer cements (RMGICs) that allow these lesions to be repaired and remineralized while removing less tooth tissue. The aim of our study was to compare the influence of aging on adhesion to sound enamel, demineralized enamel, and the healthy dentin of five RMGICs (Vitremer^®^, ACTIVA BioACTIVE Restorative, Riva LC, Ionolux^®^, and GC Fuji II LC) and fluoride release. There are currently no studies on adhesion in demineralized enamel. **Method**: A total of 1035 bovine incisors were analyzed in 45 groups of 23 teeth each. The groups were established based on three factors: time (24 h, 1 month, and 3 months); substrate (sound enamel, demineralized enamel, and healthy dentin); and type of material. In each group, 20 samples underwent shear bond strength (SBS) and fracture type analysis. Adhesive interfaces were observed in three samples from each group using field emission scanning electron microscopy (FESEM). Daily and cumulative fluoride release rates were calculated. **Results**: Adhesion improved over time on both demineralized and sound enamel. ACTIVA BioACTIVE Restorative had the highest SBS values (33.63 ± 10.69 MPa), and Vitremer^®^ had the lowest (4.10 ± 4.63). Most fractures were adhesive. Vitremer^®^ and Ionolux^®^ showed the highest daily and cumulative fluoride release rates (Vitremer daily (24 h): 225.30 ± 26.28 ppm/g; Vitremer cumulative (30 days): 635.99 ± 305.38 ppm/g; Ionolux daily (24 h): 207.59 ± 48.43 ppm/g; Ionolux cumulative (30 days): 501.21 ± 138.71 ppm/g) and ACTIVA BioACTIVE Restorative showed the lowest (ACTIVA daily (24 h): 10.50 ± 0.85; ACTIVA cumulative (30 days): 39.10 ± 2.16). **Conclusions**: ACTIVA BioACTIVE Restorative was the material with the best adhesion values on all substrates and at all times, but it showed the lowest fluoride release rates.

## 1. Introduction

In the latest report of the World Health Organization on the state of oral health worldwide, it is estimated that dental caries in permanent dentition affects 2029 million people worldwide, and caries in primary dentition affects 520 million children [1], being the most prevalent non-communicable disease affecting humanity today [2].

The composition of enamel has been studied to achieve a more resistant dental surface to any acid attack, and the composition of oral fluids, with rare exceptions, will determine whether the enamel will remain in equilibrium with the environment. Since the concentrations of calcium (Ca^2+^) and phosphate (PO_4_^3−^) in saliva are physiological and natural, the factors that influence the balance of demineralization and remineralization are the environmental pH and the presence of the fluoride ion (F^−^). The decrease in environmental pH can be considered a stressor or imbalance factor, while the presence of fluoride acts in the opposite way, reducing the impact of acid stress [3]. Organic acids resulting from the microbial metabolism of free sugars in the diet are involved in the pathogenesis of dental caries. Thus, the biofilm formed in the presence of sucrose presents low concentrations of Ca^2+^, PO_4_^3−^, and F^−^, which are critical ions in the demineralization and remineralization of enamel and dentin in the oral environment [4]. As the pH of the biofilm decreases, it reaches a point where this fluid is undersaturated with respect to the mineral on the tooth surface, and enamel dissolution occurs to maintain equilibrium. If conditions persist and the injury becomes more extensive, deeper enamel minerals and even dentin will be affected [2].

Dental caries management has evolved from “extension for prevention” to “minimally invasive” (IMO). This concept encompasses early lesion detection, radiographic classification of caries depth and progression, individual caries risk assessment, reduction in cariogenic bacteria, nonsurgical interventions, and/or a modified surgical approach that includes delayed restorations, smaller tooth preparations, the use of dental adhesives and remineralizing agents, and repair rather than replacement of failed restorations. The goal is preservation of natural tooth structure. With IMO, surgical intervention is postponed as long as possible, and treatments focus on the maximal preservation of demineralized, non-cavitated enamel and dentin [5,6].

This evolution in caries treatment is due to the wide range of materials currently available for the restoration of caries lesions. The success rate of restoration depends on the properties of the material, the level of caries risk, the condition of the affected tooth, the characteristics of the patient, and the skill of the professional in both the use of the materials and the management of the patient’s behavior in the dental chair [7]. Composite resins (CRCs) are considered the best restorative material, but due to the problems derived from polymerization shrinkage and their high sensitivity to the technique used, alternatives are sought, especially in patients with complex management and high caries recurrence rates. RMGIC is more caries-preventive than composite resin with or without fluoride [8]. Resin-modified glass ionomer cements (RMGICs) are less sensitive to manipulation than CRCs and, in addition to restoring the form and function of the teeth, they release fluoride, which can prevent the appearance of new lesions and help remineralize demineralized substrates [7].

In vitro studies on the adhesion of resin-modified glass ionomer cements (RMGICs) consistently show higher adhesion values to enamel than to dentin and a strong dependence on surface conditioning and the light-curing protocol [9,10,11,12,13]. There are no studies on demineralized enamel. Several studies have shown that enamel pre-etching/conditioning and certain surface treatments increase the shear strength of RMGICs, while more variable effects can be observed on dentin due to its moist nature and the presence of compromised dentin [9,14]. Recent studies also describe improvements in the interface and strength using complementary adhesive systems or coatings without eliminating the structural difference between enamel and dentin [14,15]. Minimal intervention techniques spare demineralized enamel, and RMGICs should be good materials for treating this substrate. Currently, in ICDAS 1, 2, 3, and 4 lesions, demineralized enamel is not removed, sealing these damaged enamel surfaces; but in addition, in cavitated lesions, minimal tissue removal is performed, leaving remains of demineralized enamel that will be potentially remineralizable [16]. To our knowledge, no specific evaluations of RMGICs adhesion on demineralized enamel have been published, which justifies the present study focused on comparing sound enamel, demineralized enamel, and dentin [17]. Although clinical trials with long-term follow-up are the intervention studies with the highest level of scientific evidence, in vitro studies are needed to control most of the variables that determine the properties of materials that cannot be controlled in vivo. Our working hypothesis is as follows:

**H1.**  *Adhesion of RMGICs to demineralized enamel is better than to sound enamel and healthy dentin.*

The main objective of this study was to study the adhesion of five RMGICs to sound enamel, demineralized enamel, and healthy dentin of bovine origin and their fluoride release over time.

## 2. Materials and Methods

This study was conducted on 1035 bovine mandibular incisors. The inclusion criteria were teeth younger than 24 months from an industrial slaughterhouse (El Cabezo, Murcia, Spain), and teeth without facial surfaces free of fractures or other defects were excluded. The study design was approved by the University of Murcia’s Experimental Bio safety Committee (CBE 198/2019) 31 May 2019.

### 2.1. Samples

**Figure 1:**
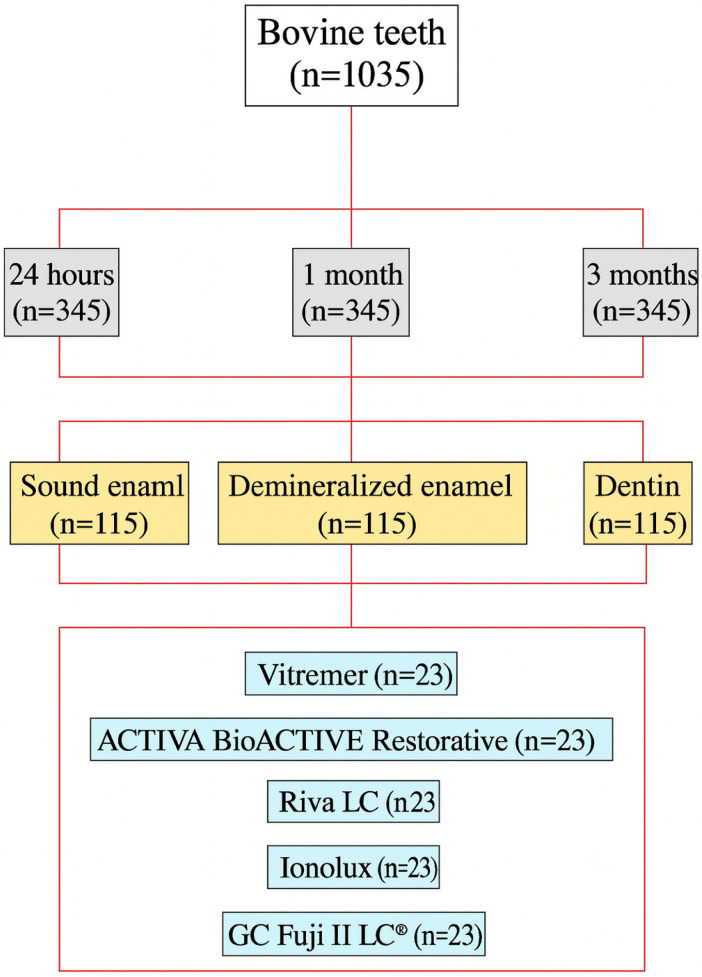
Diagram of the distribution of samples in experimental groups.

The 1035 teeth were randomly divided into experimental groups according to time (24 h, 1 month, and 3 months), substrate (sound enamel, demineralized enamel, and healthy dentin), and material (Vitremer^®^, ACTIVA BioACTIVE Restorative, Riva LC, Ionolux^®^, GC Fuji II LC^®^) (Figure 1). Randomization was performed using a list of random numbers generated by the RANDOM.ENTRE function of the Numbers program (Apple Inc., Cupertino, CA, USA). The operating researcher was blind to the randomization process. A total of 45 experimental groups consisting of 23 teeth each resulted from the process, 20 of which were used to measure shear strength, and 3 were used for field emission scanning electron microscopy (FESEM).

In the demineralized enamel groups, teeth were immersed for 48 h in a solution composed of 2.2 mM calcium chloride (CaCl_2_ 2H_2_O), 2.2 mM monosodium phosphate (NaH_2_PO_4_ 7H_2_O), and 0.05 mM lactic acid. The pH was adjusted to 4.5 with 50% sodium hydroxide (NaOH) [17]. After 48 h, the teeth were placed in an ultrasonic bath containing 90% alcohol. After 15 min, the teeth were extracted and rinsed thoroughly with distilled water, where they remained until use.

In the case of the groups where the materials under study were applied to the dentin, a diamond carving bur was used to expose it.

The materials were placed according to the manufacturer’s instructions except for Ionolux^®^, which was pretreated with GC Dentin conditioner (GC America Inc., Alsip, IL, USA) on the surface by rubbing for 20 s and washed with distilled water by spraying for 20 s. The materials Vitremer^®^, Riva LC, Ionolux^®^, and GC Fuji II LC^®^ were covered with a layer of Vitremer^®^ Finishing Gloss and polymerized for 20 s. The prepared material (Vitremer^®^, ACTIVA BioACTIVE Restorative, Riva LC, Ionolux^®^, GC Fuji II LC^®^) was condensed inside polyethylene tubes of 3 mm internal diameter and 4 mm height and polymerized with the Demi ™ Ultra LED lamp (Kerr, Orange, CA, USA). Information on the composition and batches of the materials is shown in Table S1 in Supplementary Material.

Once prepared, all samples were immersed in artificial saliva (1% carmellose sodium; 13% sorbitol; 0.12% potassium chloride; 0.084% sodium chloride; 0.005% magnesium chloride hexahydrate; 0.015% anhydrous calcium chloride; 0.017% dibasic potassium phosphate; 0.1% nigapin^®^ sodium) and placed in an oven at 37 °C for the duration of the study. The saliva was changed daily.

### 2.2. Shear Force Resistance Test (RFC)

All samples to be subjected to the RFC test were mounted in cylinders with an internal diameter of 3 cm and a height of 4 cm, immersing their roots in type IV plaster. The RFC test was performed using an AGS-1 KND Universal test machine (Schimadzu, Kyoto, Japan). The cylinders were mounted vertically. The force was applied in an incisocervical direction, perpendicular to the interface between the material and the tooth surface, using a steel bar with a 30° beveled end. The load was 1 KN, and the machine head speed was 1 mm/min. The force required to produce the debonding of the material was recorded in Newtons (N) and was converted to Megapascals (MPa) by dividing the N by the adhesion area (7.06 mm^2^), applying the formula: MPa = N/mm^2^.

### 2.3. Types of Fractures

In order to establish the type of failure that led to debonding, the fracture surfaces were examined under a ZEISS Stemi 305 stereomicroscope (Zeiss, Jena, Germany). Photographs were taken with this microscope using ZEN core v2.7 software (Zeiss, Jena, Germany). The fracture types were divided into the following four categories: cohesive enamel fracture, cohesive material fracture, adhesive fracture, and mixed fracture (partial adhesive and partial cohesive) [18].

### 2.4. Field Emission Scanning Electron Microscopy

Three samples from each group were examined using a FESEM. The roots of the bovine incisors were removed, and the crowns with adhered materials were embedded in polymethylmethacrylate. Three slices were obtained using a diamond disk (Komet, GEBR. BRASSELER GmbH & Co., Lemgo, Germany) mounted on a handpiece (Kavo Dental GmbH, Biberach, Germany) under abundant aqueous cooling. The three fragments were mounted on plates. They were placed in the Apreo S FESEM (Thermo Fisher Scientific, Waltham, MA, USA) at 2.0 kV and high vacuum.

### 2.5. Fluoride Release

The study materials were prepared according to the manufacturer’s instructions and placed inside polyethylene tubes with an internal diameter of 3 mm and a height of 4 mm. The material was condensed and polymerized using a Demi ™ Ultra LED lamp (Kerr, Orange, CA, USA). The Vitremer^®^ Finishing Gloss application step was not performed on the surface of the material. A total of 6 samples per material were prepared, removed from the polyethylene tube, and weighed on a precision balance (ENTRIS, Sartorius AG, Göttingen, Germany). The samples were then placed in test tubes containing 5 mL of deionized water. Of the 6 samples, 5 were used to calculate the cumulative fluoride release rate and 1 to calculate the daily fluoride release rate.

#### 2.5.1. Cumulative Fluoride Release Rate

Five samples per material were used. Each sample was placed in a test tube containing 5 mL of deionized water and kept in an oven at 37 °C. One sample of each material was kept for 24 h, one for 48 h, one for 7 days, one for 14 days, and one for 30 days. At the end of each time point, the sample was removed, and the test tube was kept in a freezer at −22 °C until the fluoride was measured.

#### 2.5.2. Daily Fluoride Release Rate

One sample per material was used, which was placed in a test tube containing 5 mL of deionized water at 37 °C. After 24 h, the sample was removed from the test tube and placed in another test tube containing another 5 mL of deionized water. This was repeated at 48 h, 7 days, 14 days, and 30 days. The test tubes without the sample were kept in a freezer at −22 °C until the fluoride ion determination.

#### 2.5.3. Measurement of Fluoride Ion

Fluoride ion measurements were performed using an ion-specific electrode (Orion 9609 BNWP, Thermo Fisher Scientific Inc., Waltham, MA, USA) connected to an ion analyzer (Orion EA-940, Thermo Fisher Scientific Inc., Waltham, MA, USA). The electrode was calibrated before each use with fluoride ion standard solutions ranging from 0.10 to 1.0 ppm F—by mixing 1 mL of each standard solution with 1 mL of TISAB II (Hanna Instruments, Woonsocket, RI, USA) [1.0 M acetate buffer pH 5.0, 1.0 M NaCl, and 0.4% CDTA]. Measurements were performed after all samples were thawed at room temperature and homogenized using a Classic Vortex Mixer (Velp Scientifica, Usmate Velate (MB), Italy). Test tubes were prepared with 1 mL of TISAB II and 1 mL of the test sample. The mixture was vibrated to homogenize using a Classic Vortex Mixer (Velp Scientifica, Usmate Velate (MB), Italy) for 15 s at 1200 rpm. The electrode was inserted into the test tube, and the values were allowed to stabilize. The sensor was then cleaned with distilled water. The analyzer displayed the values in millivolts (mV), which were converted to mgF/L (ppm F^−^) using calibration curves. To minimize error in the fluoride analysis, three measurements were taken from each sample, and a calibration curve was created for each measurement. The concentration per gram of material was calculated.

### 2.6. Statistical Analysis

Statistical analysis was performed using SigmaStat 3.5 (Systat Software Inc., Point Richmond, CA, USA). The sample size (n = 20 per group) was calculated using an alpha risk of 0.05 and a beta risk of 0.2 for a two-tailed comparison. The minimum difference that could be detected between two groups was considered to be 5 with a deviation of 4. The existence of 5 groups was assumed, and a loss rate of 10% was estimated.

The primary variables were shear strength and fracture type. Covariates (factors) were substrate (sound enamel, demineralized enamel, dentin), time (24 h, 1 month, 3 months), and material type (Riva LC, CG Fuji II LC^®^, Vitremer^®^, ACTIVA BioACTIVE Restorative, Ionolux^®^).

We performed a descriptive analysis of the results, which are expressed as mean ± standard deviation. When the shear strength values showed a normal distribution (Kolmogorov–Smirnov test, *p* > 0.05) and homogeneity of variance (Levene’s test, *p* > 0.05), a simple analysis of variance was performed to determine whether there were differences in the behavior of the different materials. When there were differences between groups, a Tukey test was performed to compare the groups two by two (intact enamel and dentin). When the assumptions were not met, a Kruskal–Wallis test was performed with Dunn’s test (demineralized enamel).

To determine the influence of substrate, time, and material factors on shear strength, a two-way ANOVA test was used.

To determine the association between fracture modes, substrate type and materials, Pearson’s Chi-square test was used in contingency tables.

To study the influence of time and material factors on the daily and cumulative fluoride release rate, a two-way ANOVA test was used.

Statistical significance was considered at *p* < 0.05.

## 3. Results

Not all samples made could be analyzed in the shear force resistance test since there were pre-test detachments before their analysis (Vitremer, 31.6%; ACTIVA, 0.55%; Riva LC, 7.7%; Ionolux, 9.4%; Gc Fuji, 18%). See Table S2 in Supplementary Material.

### 3.1. Resistance to Shear Forces

#### 3.1.1. Analysis at 24 h (Table S3 in Supplementary Materials)

**Figure 2:**
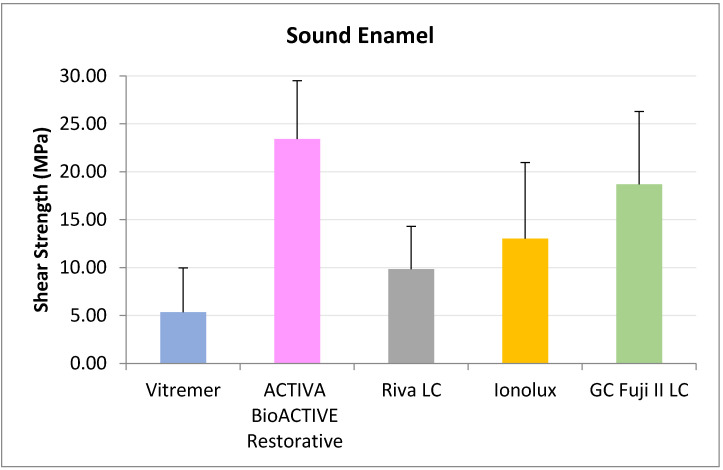
Shear strength values of glass ionomers in sound enamel at 24 h.

In sound enamel (Figure 2), ACTIVA BioACTIVE Restorative presented the highest RFC values (23.42 ± 6.10 MPa), being statistically significant compared to Riva LC (9.84 ± 4.46 MPa), Vitremer^®^ (5.35 ± 4.63 MPa), and Ionolux^®^ (13.02 ± 7.95 MPa). Vitremer^®^ presented the lowest adhesion forces, being statistically significant differences compared to ACTIVA BioACTIVE Restorative, GC Fuji II LC^®^ (18.70 ± 7.60 MPa), and Ionolux^®^ (13.02 ± 7.95 MPa). GC Fuji II LC^®^ obtained significantly higher values than Riva LC.

**Figure 3:**
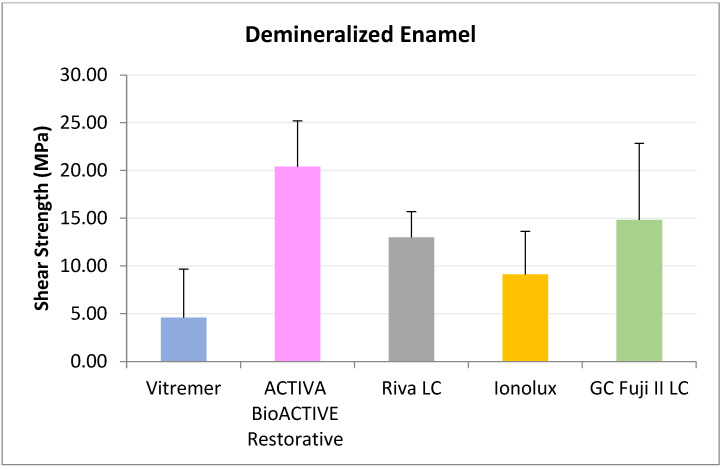
Shear strength values of glass ionomers in demineralized enamel at 24 h.

In demineralized enamel (Figure 3), ACTIVA BioACTIVE Restorative presented the highest RFC values (20.41 ± 4.79 MPa), being statistically significant compared to Riva LC (13.01 ± 2.68 MPa), Vitremer^®^ (4.63 ± 5.07 MPa), and Ionolux^®^ (9.13 ± 4.50 MPa). Vitremer^®^ presented the lowest adhesion forces, with the difference being statistically significant compared to ACTIVA BioACTIVE Restorative, Riva LC, and GC Fuji II LC^®^ (14.84 ± 7.99 MPa).

**Figure 4:**
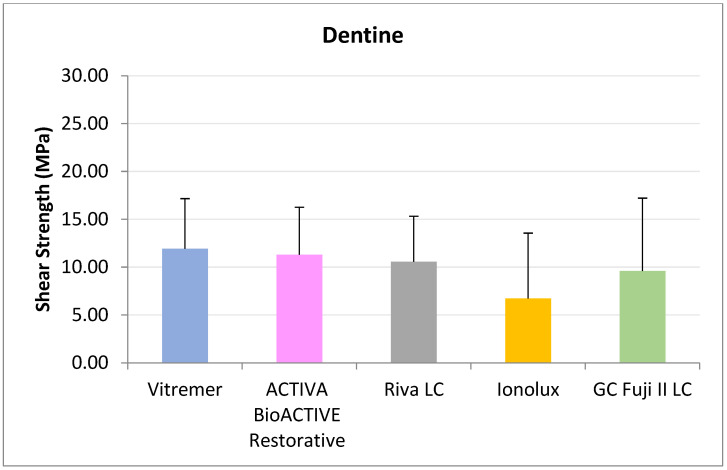
Shear strength values of glass ionomers in dentine at 24 h.

In dentine (Figure 4), all RMGICs presented similar RFC values at 24 h. There were no significant differences between the groups (*p* = 0.114).

#### 3.1.2. One-Month Analysis (Table S3 in Supplementary Materials)

**Figure 5:**
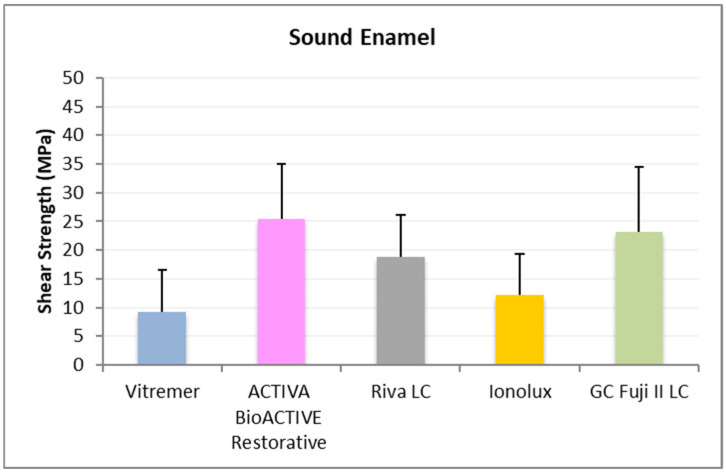
Shear strength values of glass ionomers in sound enamel at 1 month.

In sound enamel (Figure 5), ACTIVA BioACTIVE Restorative presented the highest RFC values (25.34 ± 9.67 MPa), being statistically significant compared to Vitremer^®^ (9.23 ± 7.24 MPa) and Ionolux^®^ (12.20 ± 7.14 MPa). Vitremer^®^ presented the lowest adhesion forces, with the differences being statistically significant compared to GC Fuji II LC^®^ (23.13 ± 11.35 MPa). Ionolux^®^ also presented significant differences with GC Fuji II LC^®^.

**Figure 6:**
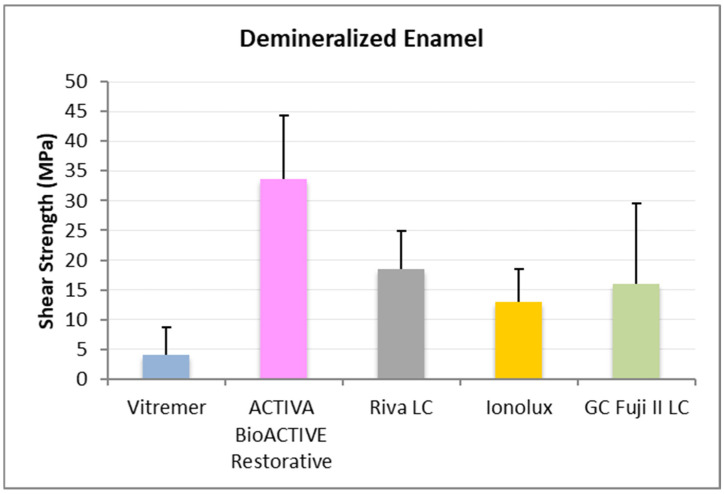
Shear strength values of glass ionomers in demineralized enamel at 1 month.

In demineralized enamel (Figure 6), ACTIVA BioACTIVE Restorative presented the highest RFC values (33.63 ± 10.69 MPa), being statistically significant compared to the rest of the materials, namely, Riva LC (18.41 ± 6.58 MPa), GC Fuji II LC^®^ (15.99 ± 13.57 MPa), Vitremer^®^ (4.10 ± 4.63 MPa), and Ionolux^®^ (12.92 ± 5.57 MPa). Vitremer^®^ presented the lowest adhesion forces, with statistically significant differences compared to Riva LC and GC Fuji II LC^®^.

**Figure 7:**
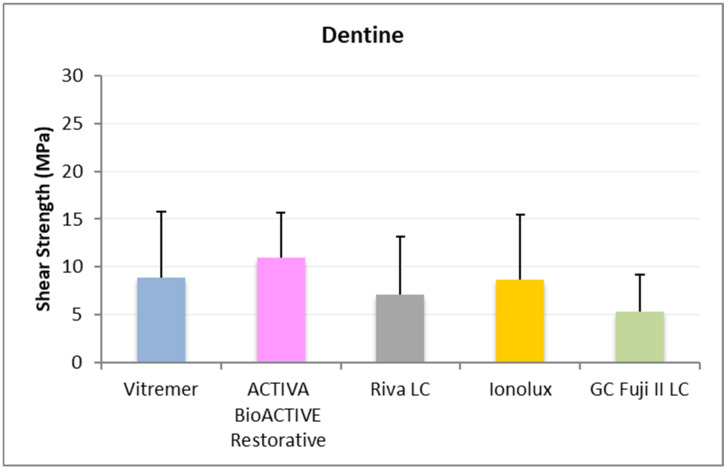
Shear strength values of glass ionomers in dentine at 1 month.

In dentine (Figure 7), ACTIVA BioACTIVE Restorative presented the highest adhesion values (11.02 ± 4.70 MPa), and the difference was significant compared to GC Fuji II LC^®^ (5.30 ± 3.93 MPa).

#### 3.1.3. Three-Month Analysis (Table S3 in Supplementary Materials)

**Figure 8:**
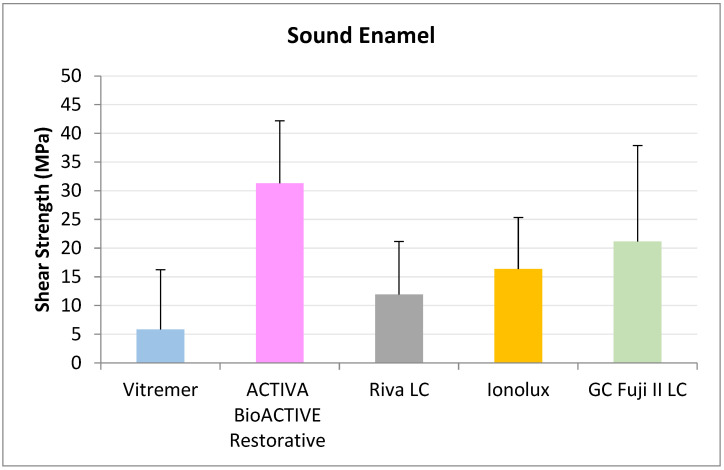
Shear strength values of glass ionomers in sound enamel at 3 months.

In sound enamel (Figure 8), ACTIVA BioACTIVE Restorative presented the highest RFC values (31.30 ± 10.38 MPa), being statistically significant compared to Riva LC (11.96 ± 9.21 MPa), GC Fuji II LC^®^ (21.15 ± 16.71 MPa), Vitremer^®^ (5.86 ± 10.87 MPa), and Ionolux^®^ (16.37 ± 8.98 MPa).

**Figure 9:**
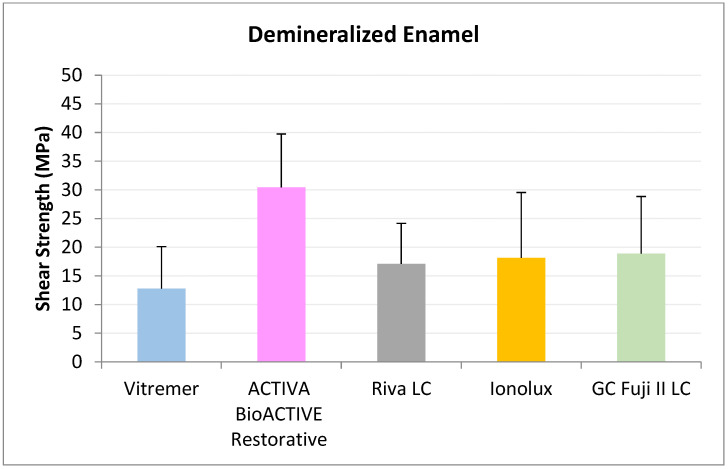
Shear strength values of glass ionomers in demineralized enamel at 3 months.

In demineralized enamel (Figure 9) ACTIVA BioACTIVE Restorative presented the highest RFC values (30.46 ± 9.28 MPa), being statistically significant compared to Riva LC (17.12 ± 7.05 MPa), GC Fuji II LC^®^ (18.91 ± 9.04 MPa), Vitremer^®^ (12.79 ± 7.34 MPa), and Ionolux^®^ (18.15 ± 11.39 MPa)

**Figure 10:**
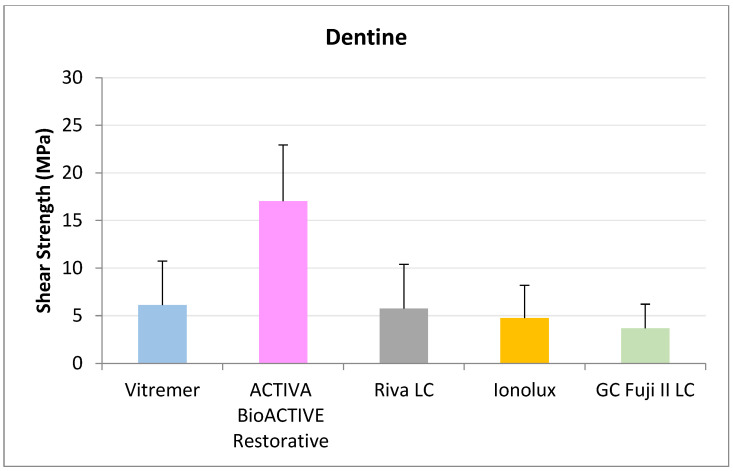
Shear strength values of glass ionomers in dentine at 3 months.

In ACTIVA dentin (Figure 10), BioACTIVE Restorative presented the highest RFC values (17.04 ± 5.89 MPa), being statistically significant compared to Riva LC (5.75 ± 4.65 MPa), GC Fuji II LC^®^ (3.68 ± 2.54 MPa), Vitremer^®^ (6.13 ± 4.61 MPa), and Ionolux^®^ (4.76 ± 3.44 MPa).

#### 3.1.4. Factorial Analysis

- Substrate vs. Time

A positive, linear interaction was observed between the substrate and time factors (*p* < 0.001). That is, time has a different influence depending on the substrate. Thus, in sound and demineralized enamel, the CFR increased over time, while in dentin, time had no influence (Table S3 in Supplementary Material).

b. Substrate vs. Material

A positive, linear interaction was observed between the substrate and material factors (*p* < 0.001). Riva LC, Ionolux^®^, and ACTIVA BioACTIVE Restorative showed lower adhesion to dentin than to normal and demineralized enamel, with no differences between them. Vitremer^®^ showed the same adhesion values regardless of the substrate. GC Fuji II LC^®^ showed differences among the three substrates (Table S3 in Supplementary Material).

c. Time vs. Material

A significant positive linear interaction between time and material (*p* = 0.02) was observed for ACTIVA BioACTIVE Restorative, which improved CFR over time. No change occurred for the other materials (Table S3 in Supplementary Material).

### 3.2. Types of Fractures

All materials showed a higher percentage of adhesive fractures at all times and substrates, but neither the type of substrate (*p* = 0.725) nor the time (*p* = 0.819) influenced the type of fracture. The influence of the material on the type of fracture was observed (*p* < 0.001). ACTIVA BioACTIVE Restorative was positively associated with cohesive enamel fractures; Riva LC and ACTIVA BioACTIVE Restorative were associated with cohesive material fractures; and Ionolux^®^ and Vitremer^®^ were associated with adhesive fractures (Table S4 in Supplementary Material).

### 3.3. Field Emission Scanning Electron Microscopy

**Figure 11:**
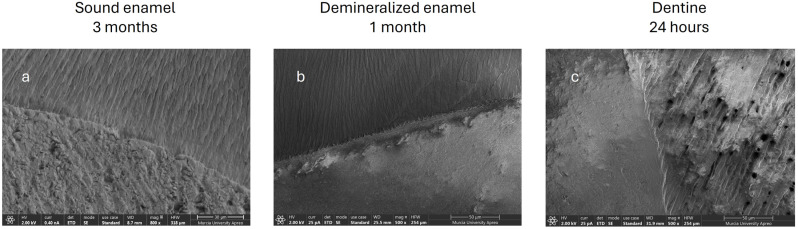
(**a**) Sound enamel, ACTIVA BioACTIVE Restorative, 3 months. (**b**) Demineralized enamel, ACTIVA BioACTIVE Restorative, 1 month. (**c**) Healthy dentin, ACTIVA BioACTIVE Restorative, 24 h.

In the images obtained with the field emission scanning electron microscope, ACTIVA BioACTIVE Restorative was observed to generate an intimate bond with all substrates at all times studied, as shown in the images (Figure 11). In Figure 11b, a perfect adhesion interface is seen; the resin microindentations facilitated by demineralization and acid etching can be seen more clearly and in greater number. In the dentin, a hybrid layer was created, and resin tags can be observed inside the dentin tubules (Figure 11c).

**Figure 12:**
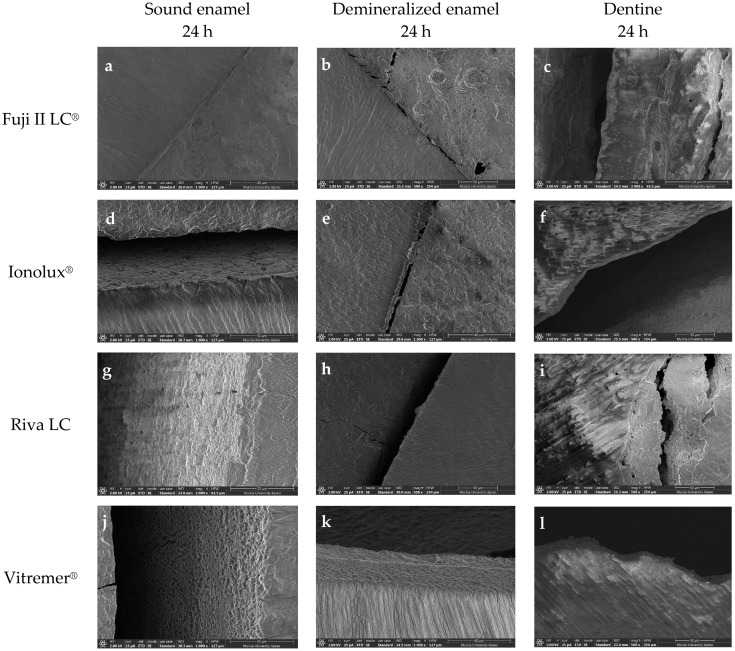
Microphotographs at 24 h obtained with the FESEM showing cohesive fractures of the Fuji II LC^®^ (**a**–**c**), Ionolux^®^ (**d**–**f**), Riva LC (**g**–**i**), and Vitremer^®^ (**j**–**l**) at the interface with all substrates.

However, the microphotographs of the remaining RMGICs did not show a bond as intimate as with ACTIVA BioACTIVE Restorative. Instead, dehydration during sample preparation, required for FESEM imaging, caused fractures at the interfaces between the materials and the substrate. In the case of Vitremer^®^ on sound enamel, the interface separation was complete (Figure 12j). However, in the other three RMGICs (Riva LC (Figure 12g), Ionolux^®^ (Figure 12d), and GC Fuji II LC^®^ (Figure 12a)), cohesive fractures of the material were observed at the interface, indicating greater adhesion strength to sound enamel. On demineralized enamel and dentin, at all times analyzed, all RMGICs also showed cohesive fractures of the material at the interface.

### 3.4. Fluoride Release Analysis

- Cumulative Fluoride Release Rate

All resin-modified glass ionomers showed continuous release of fluoride ions during 30 days of immersion in distilled water. Vitremer^®^ and Ionolux^®^ released the highest concentrations of fluoride at all times studied (Table 1).

When the interaction between material and time was studied, it was found to be statistically significant (*p* = 0.024), with cumulative fluoride ion release varying over time depending on the type of material. ACTIVA BioACTIVE Restorative showed a small, sustained release over time; Vitremer^®^ and Ionolux^®^ showed a large initial release that continuously increased up to 30 days; and Riva LC GC and Fuji II LC^®^ showed intermediate behavior (Table 1).

b. Daily Fluoride Release Rate

A statistically significant interaction was found between the time and material factors in relation to the daily fluoride release rate (*p* < 0.001) of the RMGICs that released the highest amount of fluoride at 24 h, which were Vitremer^®^ (225.30 ppm/g) and Ionolux^®^ (207.59 ppm/g). The rest of the materials had lower values: GC Fuji II LC^®^ (93.31 ppm/g), Riva LC (39.00 ppm/g), and ACTIVA BioACTIVE Restorative (10.50 ppm/g). In all cases, the release rate was highest during the first 24 h, and from then on, it decreased significantly. After 30 days, the material that released the most fluoride was Ionolux^®^, which maintained a release of 183.37 ppm/g, followed by Vitremer^®^ (130.20 ppm/g), GC Fuji II LC^®^ (82.95 ppm/g), Riva LC (68.29 ppm/g), and ACTIVA BioACTIVE Restorative (7.84 ppm/g) (Table 2).

## 4. Discussion

### 4.1. Discussion of the Method

The teeth used were of bovine origin, and although the ideal option would have been to use human teeth, there are studies that show that bovine teeth are the best substitute for research, especially in adhesion studies [19,20,21]. Likewise, although an in vivo adhesion study would better reproduce reality, it would also give highly variable responses due to the number of factors influencing the results and the complex conditions of the oral environment [14,22]. One of the advantages of in vitro research is the ability to compare the results of different published studies. Currently, the existence of multiple protocols and the lack of a standardized protocol make this comparison difficult [22,23]. In the present work, the Burke classification was used [18].

Fluoride release analysis has been performed using an electrochemical technique that uses ion-selective electrodes to provide, through the use of a multimeter, a quantitative and accurate measurement of the concentration of the dispersed ion in a liquid solution at potential. It is easy and the fastest technique for detecting fluoride ions, as well as being non-destructive and less expensive than atomic absorption spectrometry (AAS) or ion chromatography, and it can be used directly in viscous solutions. Other techniques have various drawbacks. For example, in mass spectrometry, the ion must be measured in its gaseous form; in inductively coupled plasma mass spectrometry (ICP-MS), the collaboration of highly qualified operators is required; and the fluorescence technique requires large sample quantities in the analytical procedures. Furthermore, this method of ion detection determines concentrations between 0.001 mg/L and 20,000 mg/L, and fluoride detection methods with colorimetric probes (UV–visible spectrometry) have a detection limit of 1.9 mg/L [24,25]. In the present work, values between 2.15 mg/L and 981.55 mg/L have been obtained.

### 4.2. Discussion of the Results

There were a number of samples that could not be analyzed due to pre-test failures (Table S2)—that is, samples that were already detached before they could be analyzed. The material that presented the highest number of failures was Vitremer^®^ while AC-TIVA BioACTIVE Restorer only presented one pre-test failure. The losses of the Vitremer^®^ material have already been described by other authors, such as Fritz and Uno (1996) [26], and it is also the material that presented the lowest adhesion values in our study. ACTIVA BioACTIVE Restorative presents a different adhesion system than the rest of the RMGICs, which would explain the results obtained.

Most studies on RFC have been performed on dentin [10,12,26,27,28,29,30,31,32,33], a few have been performed on enamel [9,11,26], and none have been performed on demineralized enamel. In the present work, in addition to dentin, adhesion on sound enamel and demineralized enamel has been studied since we consider that current restorations cover all three surfaces.

The working hypothesis is rejected since the adhesion on demineralized enamel is not better than on healthy enamel or on dentin. The material studied either did not demonstrate statistically significant differences between the substrates, as in Vitremer; or the adhesion value of dentin was lower than that of healthy and demineralized enamel, as in Riva LC, Ionolux; or as in ACTIVA BioACTIVE Restorative and GC Fuji II LC, materials revealed differences among the three substrates. We have observed high adhesion values at 24 h that could be explained—as noted by other authors—by a greater penetration of the material into the demineralized enamel [34]. Adhesion on sound and demineralized enamel is positively affected by the time factor—probably due to the remineralization promoted by the continuous release of fluoride, which is typical of these materials [35,36]. However, in dentin, only ACTIVA BioACTIVE Restorative showed an increase in SBS at 3 months, indicating that the stability and resistance to hydrolytic degradation of a hybrid layer resulting from a conventional Etch & Rinse adhesion procedure is superior to that generated by the adhesive procedure of a glass ionomer (polyacrylic acid).

In analyzing the results according to the material, Vitremer^®^ presents the lowest adhesion values at all times and substrates, which is similar to the results published by Fritz and Uno (1996) [26] and Glasspoole et al. (2002) [9]. The lowest value is presented in sound enamel at 24 h. Tam, Dev, and Pilliar (1995) [37] argued that this is due to the lack of microretention, which can improve with acid etching of the enamel. The highest value is presented in dentin at 24 h. We believe, like other authors [38], that this is due to the formation of the hybrid layer generated by the primer. The ACTIVA BioACTIVE Restorative material presented the best adhesion values. Our results are similar to those obtained by Nijhawan et al. (2019) [39] and similar to the RFCs of the RRCC [30,31]. Michou et al. (2018) [40] and Francois et al. (2019) [15] indicate that for this material, the use of self-etching could improve the RFC values in dentin, although they would decrease in enamel. In primary dentition, the RFC values offered by Nanavati et al. (2021) [41] and Kunal Bhatia (2022) [42] are lower due to the thinner dentin thickness and straighter dentin tubules that reduce resin tags, generally resulting in the lower adhesive capacity of the temporary teeth compared to the permanent teeth, as explained by Bordin-Aykroyd et al. (1992) [43] and Chowdhary and Reddy (2010) [44]. Regarding Riva LC, we have not found works on its adhesion to enamel. It presents similar values to the Ionolux^®^ and GC Fuji II LC^®^ materials, which are the materials with the most similar characteristics both in composition and in how they are used and handled. In all three cases, polyacrylic acid was used as an enamel conditioner to produce a soft etching pattern on the enamel and to partially remove the smear layer and allow it to penetrate both the enamel in the form of microretentions and the superficial layer of the dentin, creating a physical bonding zone of the ionomer with the dentin [29]. Imbery et al. (2013) [29] obtained lower adhesion values on dentin with Riva LC than in our study. This could be explained by the differences in the presentation of the material, which, in this work, was in powder and liquid form, and in need of handling. Although RMGICs are considered hydrophilic, they do not entirely behave well in the presence of moisture, resulting in very weak physicochemical interactions between the material and the wet, demineralized dentin; thus, the handling of the material would affect the results [10]. In the case of Ionolux^®^, we have found only one published work on its adhesion on enamel and another on dentin [11,31], with results similar to ours. Although this material does not indicate the use of polyacrylic acid for tooth preparation, we have used it to compare the conditions of use to other ionomers. Khoroushi, in his study, pretreated the enamel surface with orthophosphoric acid and found no improvement in adhesion. GC Fuji II LC^®^ can be considered the gold standard of RMGICs for comparing adhesion; we have found the largest number of works concerning GC Fuji II LC^®^—5 in enamel and 19 in dentin. In general, all of them present high adhesion values and are similar to our study (Michou et al. (2018) [40]; Lawson et al. (2012) [28]). Those studies that present lower values on enamel may do so because they used a different pretreatment than that used in our study (Harahap, Rahmi, and Winni (2022) [45]). In their studies on dentin, Swift et al. (1995) [27] likely obtained higher values because by increasing the polymerization time, they decreased the probability of errors in this step. The works of Rezvani et al. (2019) [46], Forouzanmehr et al. (2020) [47], and Moradian et al. (2021) [48] concerned materials in powder and liquid format, obtaining lower values, and this may be due to the manual preparation of the mixture.

Regarding the type of fractures, ACTIVA BioACTIVE Restorative was positively associated with cohesive enamel fractures; Riva LC and ACTIVA BioACTIVE Restorative were associated with cohesive material fractures; and Ionolux^®^ and Vitremer^®^ were associated with adhesive fractures. Even so, the fractures in our work are mostly adhesive, as in other investigations [29]. However, it is generally understood that ionomers present cohesive material fractures. If we do not take into account the percentage of the affected surface to define what type of failure occurs, almost all of the samples present some cohesive material fractures, as in the works of Francois et al. (2019) [15] and Nanavati et al. (2021) [41]. Other authors present mixed failures as the majority if more than one type appears in the same tooth [12]. The lack of unified criteria for classification is therefore the reason for this discrepancy.

In the analysis of the continuous release of fluoride, all RMGICs presented a release of fluoride ions during the 30 days in which they were immersed in distilled water. Vitremer^®^ and Ionolux^®^ that released the highest concentration of fluoride at all the times studied. When the influences of the material and time factors were studied, ACTIVA BioACTIVE Restorative had a small and sustained release over time, Vitremer^®^ and Ionolux^®^ had a large initial release that continuously increased up to 30 days, and Riva LC and GC Fuji II LC^®^ showed intermediate behavior.

In the daily fluoride release rate, there was a statistically significant interaction between the factors of time and material. The RMGICs that released the highest amounts of fluoride at 24 h were Vitremer^®^ and Ionolux^®^. They were followed by GC Fuji II LC^®^, Riva LC, and ACTIVA BioACTIVE Restorative. The release rate was highest during the first 24 h, and thereafter, it decreased significantly in all materials. After 30 days, Ionolux^®^ maintained a very high release, followed by Vitremer, GC Fuji II LC^®^, Riva LC, and ACTIVA BioACTIVE Restorative.

The release of fluoride and other ions in GICs occurs due to the presence of a counter-ion such as Na, the exchange of hydroxyl groups, and the dissolution of the ionomer in the submerged medium [13]. In bioactive glasses (Activa BIOACTIVE Restorative), the mechanism is similar, but the values found in our work, as in other investigations, are very low [49]. Fluoride release values similar to those found in our study are also found in studies such as Kosior (2023) [13] for Vitremer. However, other authors report a lower release of fluoride by Riva LC [50] and a higher release by GC Fuji II LC [32,33].

The choice of a material—analyzing adhesion and fluoride release in this case—will depend on the assessment of the caries risk in each patient. In cases of high caries risk, fluoride release will take priority, and in cases of low caries risk, adhesion will be the main property to be taken into account.

## 5. Conclusions

ACTIVA BioACTIVE Restorative exhibited the highest adhesive performance among all RMGICs evaluated on sound enamel, demineralized enamel, and dentin. Its adhesive strength improved over time. The adhesive interface generated by ACTIVA Bioactive Restorative was morphologically similar to that formed by composite resins, as indicated by the presence of cohesive fractures within enamel or dentin during bond strength testing.

All RMGICs demonstrated continuous fluoride release, with a peak observed at 24 h. Vitremer^®^ and Ionolux^®^ released higher fluoride concentrations at 24 and 48 h, as well as 7, 14, and 30 days. Vitremer^®^ showed the highest cumulative fluoride release over 30 days, whereas Ionolux^®^ exhibited the highest sustained daily release rate over time. In contrast, ACTIVA BioACTIVE Restorative released a lower but sustained amount of fluoride throughout the observation period.

Clinical relevance: This study is the first to analyze adhesion to an unfavorable substrate such as demineralized enamel. RMGICs meet the requirements of a good, minimally invasive restorative material as they provide sufficient adhesion to all three surfaces and a sustained and cumulative release of fluoride. When choosing a material from those studied in cases with low caries risk, the material of choice would be ACTIVA BioACTIVE Restorative as it presents the best adhesion values. In cases with high caries risk, the material of choice would be Ionolux as it presents acceptable adhesion values but high fluoride release.

Research and future prospects: The main limitation of this study is its in vitro nature. Randomized, controlled clinical trials are needed, as well as long-term behavioral studies. Further in vitro tests may even be performed to study the post-exfoliation interface.

## Figures and Tables

**Table 1:** Cumulative fluoride release rate.

Material	Time	Mean ± SD (ppm/g)	Median (ppm/g)
Vitremer^®^	24 h	213.17 ± 26.32	206.47
	48 h	293.08 ± 113.70	307.21
	7 d	339.93 ± 81.03	360.54
	14 d	611.10 ± 209.93	692.09
	30 d	635.99 ± 305.38	758.50
ACTIVA BioACTIVE Restorative	24 h	10.94 ± 3.27	10.09
	48 h	13.80 ± 2.19	13.74
	7 d	21.97 ± 1.18	21.80
	14 d	29.26 ± 0.69	29.37
	30 d	39.10 ± 2.16	39.74
Riva LC	24 h	43.92 ± 46.90	22.44
	48 h	67.37 ± 26.73	57.04
	7 d	177.11 ± 16.84	184.27
	14 d	224.13 ± 64.71	256.09
	30 d	295.93 ± 27.12	310.33
Ionolux^®^	24 h	197.76 ± 23.58	203.49
	48 h	229.06 ± 86.35	252.11
	7 d	315.41 ± 163.24	374.79
	14 d	448.92 ± 50.48	461.65
	30 d	501.21 ± 138.71	546.11
GC Fuji II LC^®^	24 h	82.32 ± 28.57	87.88
	48 h	115.85 ± 49.68	126.47
	7 d	145.82 ± 41.12	167.71
	14 d	191.60 ± 55.84	197.83
	30 d	371.74 ± 61.18	399.78

**Table 2:** Daily fluoride release rate.

Material	Time	Mean ± SD (ppm/g)	Median (ppm/g)
Vitremer^®^	24 h	225.30 ± 26.28	223.37
	48 h	83.33 ± 20.72	80.78
	7 d	135.05 ± 22.68	146.54
	14 d	98.96 ± 10.58	96.79
	30 d	130.20 ± 6.08	133.64
ACTIVA BioACTIVE Restorative	24 h	10.50 ± 0.85	10.26
	48 h	3.89 ± 1.78	3.17
	7 d	8.17 ± 0.40	8.16
	14 d	5.09 ± 0.35	5.11
	30 d	7.84 ± 1.92	6.76
Riva LC	24 h	39.00 ± 33.37	23.97
	48 h	18.26 ± 15.36	10.73
	7 d	72.53 ± 11.80	78.26
	14 d	62.42 ± 2.57	63.70
	30 d	68.29 ± 3.27	69.55
Ionolux^®^	24 h	207.59 ± 48.43	230.94
	48 h	154.08 ± 32.99	158.65
	7 d	215.13 ± 42.14	232.57
	14 d	181.24 ± 21.52	186.94
	30 d	183.37 ± 19.71	183.56
GC Fuji II LC^®^	24 h	93.31 ± 16.59	93.09
	48 h	28.29 ± 6.88	28.31
	7 d	60.98 ± 4.80	58.55
	14 d	59.16 ± 3.21	60.72
	30 d	82.95 ± 5.66	81.94

## Data Availability

The raw data supporting the conclusions of this article will be made available by the authors on request.

## Supplementary Materials

The following supporting information can be downloaded at https://www.mdpi.com/article/10.3390/jcm14207166/s1. Table S1. Composition of materials according to the safety data sheet published by the manufacturers; Table S2. Pre-test failures; Table S3. Shear strength values of glass ionomers in sound enamel, demineralized enamel and dentin at 24 h, 1 month and 3 months; Table S4. Types of glass ionomer fractures in sound enamel, demineralized enamel, and healthy dentin.

## Abbreviations

The following abbreviations are used in this manuscript:

MID	Minimally invasive dentistry
RMGICs	Resin-modified glass ionomer cements
SBS	Shear bond strength
FESEM	Field emission scanning electron microscopy
IMO	Minimally invasive dentistry
CRCs	Composite resins
RFC	Shear force resistance test
MPa	Megapascals
