# Supplementary material for: Time-Dependent Adhesion and Fluoride Release of Resin-Modified Glass Ionomer Cements on Demineralized Enamel, Sound Enamel and Dentine
Source: J Clin Med. 2025 Oct 11;14(20):7166. doi: 10.3390/jcm14207166 (PMC12565266; doi:10.3390/jcm14207166)
Supplement: Supplementary file 1 [file jcm-14-07166-s001.zip › jcm-3890010-supplementary.pdf]

Supplementary material for "Time-Dependent Adhesion and Fluoride Release of Resin-Modified Glass Ionomer Cements on Demineralized Enamel, Sound Enamel and Dentine"

Table S1. Composition of materials according to the safety data sheet published by the manufacturers.

Product	Lot No.	Composition	w/w %
First Vitremer ®	N907817<br>N818172<br>N967006	2-Hydroxyethyl methacrylate.	44-55
		Ethyl alcohol.	35-45
		Copolymer of Itaconic and Acrylic Acids	10-15
		Diphenyliodonium hexafluorophosphate.	<1
Vitremer ® Liquid	N907662<br>N977402<br>N972914	Polymer of 2-propenoic acid and methylenebutanedioic acid.	45-50
		Non-hazardous ingredients.	25-30
		2-Hydroxyethyl methacrylate.	15-25
		Diphenyliodonium hexafluorophosphate.	<1
Vitremer ® Powder	N911066<br>N930416<br>N969444	Glass powder, surface modified with 2-propenoic acid, 2-methyl-.3-(trimethoxysilyl)propyl ester (2530-85-0), bulk material.	90-99.9
		Dipotassium peroxodisulfate.	<0.15
Finishing Gloss Vitremer ®	N909562<br>N886682<br>N750696<br>N967005	2,2'-Ethylenedioxydiethyl dimethacrylate (TEGDMA)	40-60
		(1-methylethylidene)bis[4,1-phenylenexy(2-hydroxy-3,1-propanedyl)] bismethacrylate	40-60
		Triphenylstibine	<1
		4-(Dimethylamino)phenethyl alcohol	<0.5
		Hydroquinone	<0.1
ACTIVA BioACTIVE Restorative	183124<br>181029	Mixture of diurethane and other methacrylates with modified polyacrylic acid	44.6
		Amorphous silica	6.7
		Sodium fluoride	0.75
Riva Light Cure	170309	Compartment 1	
		Polyacrylic acid	15-25
		Tartaric acid	1-5
		2-hydroxyethyl methacrylate	20-25
		Dimethacrylate cross-linker	10-25
		Acid monomer	10-20
		Compartment 2	
		Fluoroaminosilicate (glass powder)	95-100
Ionolux ®	183124	Polvo	
		Fluorosilicate glass	50-100
		Polyacrylic acid	2.5-5
		Liquid	

		2-hydroxyethyl methacrylate	25-50
		Polyacrylic acid	10-25
		Glycerindimethacrylate	10-25
		Urethanedimethacrylate	10-25
GC Fuji II LC ®	180622<br>180718A<br>180809A	2-hydroxyethyl methacrylate (HEMA)<br>Polybasic carboxylic acid*<br>Urethane dimethacrylate (UDMA)<br>Dimethacrylate*<br>*The substance is a trade secret	25-50<br>5-10<br>1-5<br>1-5
Dentaflux Acid	202818<br>020818<br>021218	Orthophosphoric acid<br>Receiving	37<br>Up to 100
Prime & Bond Active ™	1702000427<br>1706000231<br>1702000427<br>1701000256	Bisacrylamide1<br>Propan-2-ol<br>10-Methacryloyl-oxidicyl- dihydrogenophosphate<br>Bisacrylamide 2<br>Dipentaerythritol phosphate pentaacrylate<br>4-(dimethylamino)benzonitrile	25-50<br>10-<25<br>10-<25<br>2.5-<10<br>2.5-<10<br>0.1-<1
Riva Conditioner	170309	Polyacrylic acid	25-30%
GC Dentin conditioner	1807101	Polyacrylic acid	5-10%

w/w %: percentage by weight.

Table S2. Pre-test failures.

	Sound Enamel			Demineralized Enamel			Dentine		
	24h	1month	3months	24h	1month	3months	24h	1month	3months
Vitremer®	8	12	12	6	6	3	0	3	7
ACTIVA BioACTIVE Restorative	0	0	0	0	0	0	0	0	1
Riva LC	1	0	1	0	0	0	4	2	6
Ionolux ®	0	0	3	0	0	6	5	1	2
GC Fuji II LC ®	0	2	11	1	1	5	1	1	11

Table S3. Shear strength values of glass ionomers in sound enamel, demineralized enamel and dentin at 24 hours, 1 month and 3 months.

	24 Hours						1 Month						3 Months					
	Sound Enamel		Demineralized Enamel		Dentine		Sound Enamel		Demineralized Enamel		Dentine		Sound Enamel		Demineralized Enamel		Dentine	
	MPa	$p < 0.05$	MPa	$p < 0.05$	MPa	$p < 0.05$	MPa	$p < 0.05$	MPa	$p < 0.05$	MPa	$p < 0.05$	MPa	$p < 0.05$	MPa	$p < 0.05$	MPa	$p < 0.05$

Vitremer ®	5.35 ± 4.63 a	4.63 ± 5.07 a	11.93±5.23	–	9.23±7.24 a	4.10±4.63 a	8.92±6.84	5.86±10.38 a	12.79±7.34 a	6.13±4.61 A
ACTIVA BioACTIVE Restorative	23.42 ± 6.10	20.41 ± 4.79	11.30±4.96	–	25.34±9.67	33.63±10.69	11.02±4.70	31.30±10.87	30.46±9.28	17.04 ±5.89
Riva LC	9.84 ± 4.46 a	13.01 ± 2.68 a, b	10.58±4.74	–	18.83 ±7.23	18.41 ± 6.58 a, b	7.11 ±6.02	11.96 ±9.21 a	17.12 ±7.05 a	5.75 ±4.65 A
Ionolux ®	13.02 ± 7.95 a, b	9.13 ± 4.50 a	6.73±6.83	–	12.20±7.14 a	12.92±5.57 a	8.64±6.87	16.37±8.98 a	18.15±11.39 a	4.76±3.44 A
GC Fuji II LC®	18.70 ± 7.60 b, c	14.84 ± 7.99 b	9.60±7.62	–	23.13±11.35 b, d	15.99±13.57 a, b	5.30±3.93 a	21.15±16.71	18.91±9.04 a	3.68±2.54 A
	a vs ACTIVA BioACTIVE Restorative; b vs Vitremer®; c vs Riva LC				a vs ACTIVA BioACTIVE Restorative; b vs Vitremer®; d vs Ionolux®			a vs ACTIVA BioACTIVE Restorative; b vs Vitremer®; d vs Ionolux®		

Table S4. Types of glass ionomer fractures in sound enamel, demineralized enamel, and healthy dentin.

Cluster			Cohesive Enamel Fracture	Cohesive Fracture Material	ofAdhesive Fracture	Mixed Fracture
24 hours	Demineralized enamel	Vitremer ®	0% (0/10)	0% (0/10)	100% (10/10)	0% (0/10)
		ACTIVA BioACTIVE Restorative	5% (1/20)	25% (5/20)	65% (13/20)	5% (1/20)
		Riva LC	0% (0/18)	11% (2/18)	89% (16/18)	0% (0/18)
		Ionolux®	0% (0/18)	0% (0/18)	100% (18/18)	0% (0/18)
		GC Fuji II LC ®	0% (0/19)	5% (1/19)	84% (16/19)	11% (2/19)
	Sound enamel	Vitremer ®	0% (0/12)	0% (0/12)	100% (12/12)	0% (0/12)
		ATIVA BioACTIVE Restorative	5% (1/20)	15% (3/20)	80% (16/20)	0% (0/20)
		Riva LC	0% (0/19)	0% (0/19)	100% (19/19)	0% (0/19)
		Ionolux®	0% (0/20)	0% (0/20)	100% (20/20)	0% (0/20)
		GC Fuji II LC ®	0% (0/20)	0% (0/20)	100% (20/20)	0% (0/20)
	Dentine	Vitremer®	0% (0/20)	5% (1/20)	95% (19/20)	0% (0/20)
		ACTIVA BioACTIVE Restorative	0% (0/20)	0% (0/20)	100% (20/20)	0% (0/20)
		Riva LC	0% (0/16)	25% (4/16)	69% (11/16)	6% (1/16)
		Ionolux®	0% (0/15)	0% (0/15)	100% (15/15)	0% (0/15)
		GC Fuji II LC®	5% (1/19)	0% (0/19)	90% (17/19)	5% (1/19)
1 mes	Demineralized enamel	Vitremer®	0% (0/14)	0% (0/14)	100% (14/14)	0% (0/14)
		ACTIVA BioACTIVE Restorative	5% (1/20)	0% (0/20)	95% (19/20)	0% (0/20)
		Riva LC	0% (0/20)	10% (2/20)	85% (17/20)	5% (1/20)

		Ionolux®	0% (0/20)	0% (0/20)	95% (19/20)	5% (1/20)
		GC Fuji II LC ®	0% (0/19)	0% (0/19)	959% (18/19)	5% (1/19)
		Vitremer ®	0% (0/8)	0% (0/8)	100% (8/8)	0% (0/8)
	Sound enamel	ATIVA BioACTIVE Restorative	5% (1/20)	5% (1/20)	90% (18/20)	0% (0/20)
		Riva LC	0% (0/20)	10% (2/20)	90% (18/20)	0% (0/20)
		Ionolux®	5% (1/20)	0% (0/20)	95% (19/20)	0% (0/20)
		GC Fuji II LC®	0% (0/18)	6% (1/18)	94% (17/18)	0% (0/18)
		Vitremer®	0% (0/17)	0% (0/17)	100% (17/17)	0% (0/17)
	Dentine	ACTIVA BioACTIVE Restorative	0% (0/20)	5% (1/20)	95% (19/20)	0% (0/20)
		Riva LC	0% (0/18)	11% (2/18)	89% (16/18)	0% (0/18)
		Ionolux®	0% (0/19)	11% (2/19)	90% (17/19)	0% (0/19)
		GC Fuji II LC ®	0% (0/19)	0% (0/19)	100% (19/19)	0% (0/19)
		Vitremer ®	0% (0/17)	0% (0/17)	100% (17/17)	0% (0/17)
	Demineralized enamel	ATIVA BioACTIVE Restorative	0% (0/20)	10% (2/20)	90% (18/20)	0% (0/20)
		Riva LC	0% (0/20)	0% (0/20)	100% (20/20)	0% (0/20)
		Ionolux®	0% (0/14)	0% (0/14)	100% (14/14)	0% (0/14)
		GC Fuji II LC ®	7% (1/15)	0% (0/15)	93% (14/15)	0% (0/15)
		Vitremer ®	0% (0/8)	0% (0/8)	100% (8/8)	0% (0/8)
3 months	Sound enamel	ATIVA BioACTIVE Restorative	0% (0/20)	5% (1/20)	90% (18/20)	5% (1/20)
		Riva LC	0% (0/19)	5% (1/19)	95% (18/19)	0% (0/19)
		Ionolux®	0% (0/17)	0% (0/17)	100% (17/17)	0% (0/17)
		GC Fuji II LC®	0% (0/9)	0% (0/9)	89% (8/9)	11% (1/9)
		Vitremer®	0% (0/13)	0% (0/13)	100% (13/13)	0% (0/13)
	Dentine	ACTIVA BioACTIVE Restorative	11% (2/19)	16% (3/19)	74% (14/19)	0% (0/19)
		Riva LC	0% (0/14)	7% (1/14)	93% (13/14)	0% (0/14)
		Ionolux®	0% (0/18)	0% (0/18)	100% (18/18)	0% (0/18)
		GC Fuji II LC ®	0% (0/9)	0% (0/9)	100% (9/9)	0% (0/9)
